# Case report: Clinical lycanthropy in Huntington's disease

**DOI:** 10.3389/fpsyt.2023.1089872

**Published:** 2023-01-27

**Authors:** Nick Medford, Natasha Sigala

**Affiliations:** ^1^Department of Neuropsychiatry, South London and Maudsley NHS Foundation Trust, London, United Kingdom; ^2^Department of Neuroscience, Brighton and Sussex Medical School, University of Sussex, Brighton, United Kingdom

**Keywords:** lycanthropy, Huntington's disease, delusions, psychosis, zoanthropy, werewolf, intermetamorphosis

## Abstract

We describe the case of a patient diagnosed with Huntington's disease (HD), who, following a two-year history of anxiety with obsessional preoccupations, developed psychosis with clinical lycanthropy: a prominent delusional idea that he was a werewolf. Although there was no benefit from various antidepressants and antipsychotics, there was remarkable improvement of his symptoms following prescription of Clozapine. His choreiform movement disorder also improved as his mental state settled. Although some reported cases of clinical lycanthropy are related to neurological conditions, this is the first case in a patient with HD. We also discuss the relevance of cultural and personal factors in the expression of a delusion that incorporates disgust, and the potential role of somatosensory aberrations and misidentification of self.

## 1. Introduction and case description

A 63-year-old white British man with a diagnosis of Huntington's disease (HD) was admitted to an inpatient mental health unit, and transferred to the specialist inpatient neuropsychiatry unit 3 weeks later. He had an approximately 2-year history of anxiety with obsessional preoccupations, progressing to psychosis, and his condition had not improved despite various antidepressants and antipsychotics. On admission, he was in a state of near-constant mental and physical agitation, with prominent choreiform movements affecting his upper body, and repeatedly stated that he was becoming a werewolf. He was preoccupied with this and a range of other transgressive and apocalyptic ideas, stating that he had an urge to strangle his wife and eat her body, also to defecate in a church, and that God had died (despite also saying he did not believe in God). He had not acted on these ideas apart from one occasion prior to admission when he had placed his hands around his wife's neck, but had not exerted any pressure; his wife stated that she did not feel in danger during this incident.

Psychological difficulties began about 2 years prior to this admission. At that time, he was living in France, and had become increasingly anxious, developing what his wife described as “mood swings”, becoming unusually irritable at times, interspersed with periods of low mood. He was prescribed Paroxetine which initially appeared to be helpful. About a year after this, he again became anxious and began to develop unusual preoccupations (for example, around feces) as well as a compulsion to strangle his wife. He was prescribed Olanzapine which was gradually increased over several weeks to a dose of 20 mg per day, but his wife reported that his mental state and sleep continued to worsen, and he was admitted to a psychiatric unit in France for 1 week. In addition to the above symptoms, at this time he was also describing odd electric-shock type sensations which moved from the left side of his head to his left arm ‘as if my body was freezing'. His symptoms continued to worsen and after another 2 months he had a further 4-week psychiatric admission, now saying he had the sense that he was an animal, which then developed into the idea that he was turning into a werewolf. His preoccupations with feces and strangulation intensified around this time. In France he was prescribed typical antipsychotics (Loxapine and later Cyamemazine), in combination with Olanzapine. His wife did not think this was beneficial. Mianserin was also commenced which was reported to have helped with sleep, while he was recorded as having had a paradoxical reaction to benzodiazepines, which were avoided thereafter. Some of the drugs prescribed in France reflect differences in treatment approaches, as these are only rarely prescribed in the UK.

He and his wife subsequently returned to the UK. His agitation, and the intensity of his obsessional preoccupations, and belief that he was becoming a werewolf, increased, leading to an emergency admission as above, initially to a general adult psychiatry ward, with transfer to a specialist neuropsychiatry unit after 3 weeks.

Background history was that his mother had been diagnosed with HD when she was 50, and had died at the age of 60. He recounted distressing memories from his teenage years of his mother being in a state of aggressive agitation. He himself received his HD diagnosis at the age of 58, following genetic testing while he was asymptomatic, thinking it would prove that he was not a carrier. This genetic testing revealed a 41 CAG repeat. He did not smoke, or use illicit substances, and drank alcohol only occasionally. This presentation was his first contact with psychiatric services in the UK.

## 2. Diagnostic assessment and therapeutic interventions

**Figure 1:**
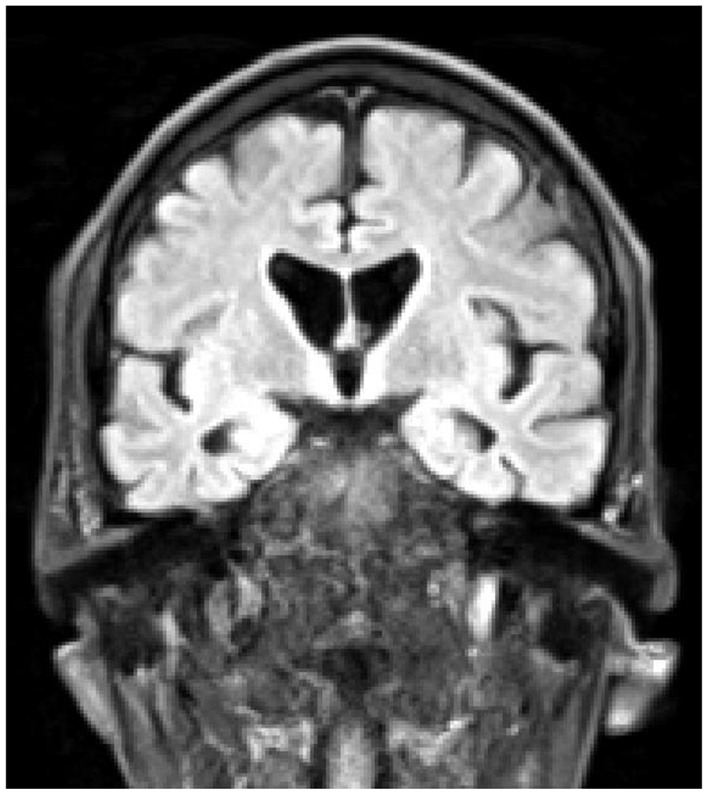
Coronal T1-weighted MRI scan, demonstrating atrophy of the caudate nuclei, and enlargement of the lateral ventricles.

At the time of this admission he was not on any antipsychotic medication, but was started on Quetiapine, and at the time of transfer to the specialist neuropsychiatry unit his prescription was slow-release Quetiapine 200 mg per day, Mianserin 10 mg at night, plus Promethazine 25 mg as required for agitation, and Lactulose and Sodium Docusate as required for constipation. His belief that he had become a werewolf was expressed frequently and he would look in a mirror and state that his physical appearance had changed. At this point he was also preoccupied with his bowel function, stating repeatedly that he was “completely blocked”, and that this was somehow connected with the death of God and the imminent end of the universe. He also stated that he was the Devil, that he was physically changing and shrinking, and that he believed he would die soon because his internal organs were becoming exposed. He was able to acknowledge that these ideas did not hang together, and felt unable to explain this, even to himself. On initial physical examination he had prominent choreiform movements of his upper body, but normal tone, power, sensation and brisk reflexes. Cognitive examination was challenging as his level of agitation made it hard for him to engage with testing, but no gross cognitive deficits were identified. He was however apparently unaware of his choreiform movements. A structural MRI brain scan showed unusually small caudate nuclei and widening of the lateral ventricles, consistent with a diagnosis of HD (Figure 1).

Early in his admission to the neuropsychiatry unit, there were a few occasions when he lunged at members of staff with his hands poised to take hold of their necks, but would pull away before making contact and apologize. His agitation and outbursts of aggression necessitated 1:1 nursing and detention under the Mental Health Act, as he was felt to be a risk to others. His medication compliance was variable, and there was no evidence of improvement when taking Quetiapine despite this medication being increased to 300 mg bd and prescribed at this dose for a further 4 weeks. A decision was made to switch to Clozapine and this was slowly titrated to a dose of 450 mg per day in divided doses. Initially the necessary blood monitoring was very challenging as he would refuse blood tests, saying that the needle was larger than his arm, or that there was no blood in his veins, or that there was no point sending his blood for analysis as he no longer had human blood. It was necessary for medical staff to spend considerable time calming and reassuring him before he would agree to blood tests. As the dose of Clozapine was increased and his mental state improved, this became more straightforward.

Soon after the introduction of Clozapine he became more settled and it became easier to engage him in conversation and ward activities. It was felt that there was a depressive element to his psychosis, as evidenced by nihilistic delusions, and Mianserin was switched to Citalopram, initially 10 mg per day, increasing to 20 mg per day after 1 week.

There were some adverse effects of Clozapine, with an initial worsening of his constipation necessitating an increase in laxative use, and hypersalivation, which fortunately settled without the need for specific treatment.

Over the following weeks, his mental state improved as Clozapine was slowly up-titrated. His unusual ideas and beliefs became less prominent or disappeared altogether. He was no longer agitated or aggressive, and was euthymic, largely free of anxiety, and able to reflect on his experiences. The Mental Health Act detention was rescinded, with him remaining on the ward as a voluntary patient. A period of trial leave from the ward, staying with his wife, went well with no concerns raised, and he was discharged soon after. Notably, as he improved, his movement disorder also became much less prominent, and by the time of discharge this was only evident as occasional subtle involuntary jerks of his upper body. At this stage he was able to engage more fully in formal cognitive testing, which demonstrated above average IQ with no evidence of cognitive deficit.

Approximately 4 years following discharge, he is on no psychotropic medication but remains relatively well, with no recurrence of psychotic symptoms and only mild movement disorder, consistent with his genetic testing, which would suggest he will be relatively mildly affected by HD. He is living independently with his wife and is able to self-manage all activities of daily living, and pursue his interest in gardening.

## 3. Discussion

Huntington's disease (HD) is an inherited genetic progressive neurodegenerative disease with cognitive, motor and psychiatric symptoms, caused by a mutant protein, huntingtin, which results from an unstable expanded trinucleotide CAG repeat on chromosome 4. In the normal population the CAG repeat length ranges from 10 to 35, whereas in patients with HD it ranges from 36 to 121, with complete penetrance occurring from 39 repeats (1), the number of repeats being negatively correlated with the age of onset of HD (2), and positively correlated with rate of caudate atrophy (3). At protein synthesis, these abnormal CAG repeats result in a polyglutamine chain being incorporated into the huntingtin protein, producing a mutant form which accumulates in cells and is neurotoxic. The medium spiny neurons of the striatum are particularly susceptible, and atrophy of the caudate nuclei is the characteristic radiological finding (1).

Clinically, HD is characterized by the triad of the typical movement disorder, subcortical dementia, and a positive family history, with onset of overt symptoms usually occurring in the fourth or fifth decade. Psychosis occurs in 3–11% of patients with HD (4), usually as a relatively late manifestation. The decision to use Clozapine, despite this being an off-license indication, was based on the fact that multiple antipsychotic medications had already been tried without benefit, and there are published cases in which treatment-resistant psychosis in HD responded to Clozapine (5–7). In the last two of these case reports, the authors note that good response to Clozapine was seen only at doses of 425 mg and 450 mg per day respectively, higher doses than those used in earlier reports (7). This latter (450 mg per day) is the same dose on which our patient was stabilized, although in our case a gradual improvement in mental state was noted throughout the up-titration. There is also evidence that Clozapine may have a role in ameliorating the movement disorder of HD (8), although an open-label trial suggested these benefits are modest at best (9).

It is now well recognized that subtle cognitive, emotional, and behavioral changes often predate the onset of other symptoms of HD, sometimes by decades (1). Commonly these changes are irritability, cognitive rigidity and stubbornness, depression and anxiety with obsessional preoccupations, and apathy (10). These features may be severe enough to attract a diagnosis of organic personality disorder. In our case, there was evidence of these features (aside from apathy) being present for at least 2 years prior to the development of frank psychosis. The psychosis itself was poorly systematized, as is commonly observed in HD-associated psychosis, with a range of ideas and beliefs that did not hang together, unlike the elaborate delusional systems frequently encountered in schizophreniform illness. In these respects, this case illustrates some quite typical features of mental and behavioral changes associated with HD. The highly unusual feature of our case is clinical lycanthropy: the patient's belief that he was transforming into a werewolf.

While clinical lycanthropy (the delusion of transformation into a wolf, from Greek lykos, “wolf” and anthropos, “human”) is rare, the wider cultural idea of the werewolf has an extensive legendary and literary history, e.g. (11–13). The Ancient Greeks worshiped Zeus Lycaeus, who transformed Lycaon, the cruel King of Arcadia (a region plagued by wolves) as punishment for serving the god a dinner of human flesh, according to Ovid's Metamorphoses, Book I (14). In Ancient Egypt, Anubis, the god of Death and the Underworld has the head of a jackal, which is an African golden wolf (15). Anubis is also represented with the lunar disk, as a symbol of resurrection and rebirth (16), a theme repeated in the Byzantine iconography of St Cristopher (17), potentially referring to an ancient association of werewolves with the moon (15). The werewolves of antiquity symbolized moral shortcomings, while later accounts in Scripture implied the interference of satanic forces in human affairs (13). During the Inquisition, reports of werewolves reached ‘moral panic' proportions: 30,000 were supposedly recorded in France alone between 1520 and 1630 (18). Why the idea of the werewolf should have recurred across so many cultures and eras is an intriguing question. A creature that is part-human, part-wolf can readily be seen as symbolizing the struggle between the civilized aspect of the human, with the accompanying obligations to observe social norms, and the animalistic, instinctual aspect, which chafes against such restrictions. A frequent feature of werewolf stories is that full transformation into wolf form is associated with frenzied violence and sexual activity, followed by guilt and self-loathing once a human form is regained. These themes may have explanatory power when it comes to cases of werewolf delusions: clinical lycanthropy. Fahy (12) summarizes case studies of clinical lycanthropy from the nineteenth and twenteeth centuries as compatible with patients' ≪ perception of themselves as evil, disgusting or guilty ≫, and argues that the powerful and evocative image of a werewolf's aggressive, cannibalistic qualities can be related to delusions characterized by guilt, sinfulness and disgust (12). Delusions are influenced by culture (19, 20), types of family relationships, and concepts of the self (15, 21, 22). We explore the relevance of these factors to our case further below. It has been suggested that lycanthropy is best classified as a Delusional Misidentification Syndrome (DMS), involving a global misidentification of self (23, 24). Another idea, discussed further below, considers lycanthropy as a form of cenesthopathy (pathological bodily sensation), developing as a result of somatic hallucinations and somatosensory aberrations (24, 25). About 24 cases of clinical lycanthropy have been reported in the medical literature between 1852 and 2020 (15); this case report is the first for a patient with HD.

To understand why this patient should have developed this particular delusion, and some of the other unusual ideas he expressed, it is necessary to move beyond descriptive labels such as “psychosis” and consider the phenomenology in the light of his subjective experience and the ideas outlined above. Although he was apparently unaware of his movement disorder, he nevertheless described strange and unpleasant physical sensations, a common experience in HD, and expressed a range of delusional ideas relating to bodily changes. In addition, he stated he had always had a horror of developing HD, in part because of painful memories of his mother's psychological deterioration during his childhood. He had reached the age of 58, apparently asymptomatic, when he underwent genetic testing to confirm to himself that he did not carry the HD gene, and was shocked to find that he did. It seems plausible that his sense that he was turning into a werewolf – something fiendish, monstrous – was rooted in this terror of the disease, coupled with some distorted perceptions of the bodily changes linked to the disease process. Some of his other symptoms, such as his sense that he was shrinking, or his apparently perceiving a hypodermic needle as larger than his arm, further support the idea that sensory misperceptions were a key element in the development of symptoms. Here the idea of “secondary clinical lycanthropy” (25) is relevant – the notion that lycanthropic delusions can arise from somatic hallucinations and/or alterations in the sense of physical identity in people with psychotic illnesses, principally schizophrenia. In our patient, there were actual, not merely hallucinatory, changes in his physical state, and a knowledge that he had a serious progressive disease of which he had a particular horror. The caudate nuclei (atrophied in this patient) are part of the striatum, which, among other functions, regulates feelings of reward, pleasure, and aversion to negative stimuli e.g. (26), as well as disgust processing, including moral violations (27, 28). Abnormal perception of disgust, as well as self-related disgust, has been specifically suggested as a contributing element in the development of clinical lycanthropy (29). This combination of factors, all readily applicable to our case, would seem to provide fertile ground for the development of clinical lycanthropy, based on misattribution of bodily sensations and a fear of becoming unwell, negatively altered, and socially unacceptable through the progression of HD.

The consideration of phenomenology, and of the meaning of symptoms, should always be at the heart of any attempt to understand and formulate psychiatric presentations, but it is all too easy for this principle to be abandoned in the context of organic conditions such as HD, where the presence of well-established genetic and biochemical abnormalities may seem to provide sufficient explanation for symptoms. However, knowledge of the biomedical basis of disease should not discourage a psychodynamic approach – rather these two types of knowledge should be complementary. In this case, while the underlying diagnosis of HD was already known, understanding the content of the patient's psychosis depends on a psychological formulation based on the patient's particular life history and subjective experience. This blending of scientific knowledge with phenomenological insight is the essence of neuropsychiatric formulation and practice.

## Data availability statement

The original contributions presented in the study are included in the article/Supplementary material, further inquiries can be directed to the corresponding author.

## Ethics statement

Ethical review and approval was not required for the study on human participants in accordance with the local legislation and institutional requirements. The patients/participants provided their written informed consent to participate in this study. Written informed consent was obtained from the individual(s) for the publication of any potentially identifiable images or data included in this article.

## Author contributions

NM oversaw the clinical care of the patient and prepared the case description. NS made important conceptual contributions and added theoretical material to the Discussion section. Both authors contributed to the article and approved the submitted version.
